# HOW DO PEOPLE PERCEIVE THEIR OWN AGE?—SELF-REFLECTION ON HEALTH STATUS

**DOI:** 10.1093/geroni/igad104.2707

**Published:** 2023-12-21

**Authors:** Hyojin Choi, Kristin Litzelman

**Affiliations:** UW-Madison Institute on Aging, Madison, Wisconsin, United States; University of Wisconsin-Madison, Madison, Wisconsin, United States

## Abstract

Subjective age -- how old you feel -- is a simple, but inclusive indicator of how people experience their own aging. This hypothesis-generating study sought to assess how objective and subjective indicators of well-being differ by subjective age, in order to progress our understanding of how this metric internalizes physical and psychological information. Data were drawn from the Midlife in the United States in 2004-2006 (MIDUS 2). The sample represented adults aged 50 years and over (n=612) and was divided into two groups: 1) felt younger than chronological age, and 2) felt the same or older than chronological age. Nearly four in five older adults reported feeling younger than their chronological age. Chi-square statistics showed that subjective indicators differ by subjective age. Specifically, those that felt the same/older than their chronological age were more likely to have depression or functional limitations, and report poor health status than were feeling younger group. Interestingly, bio-marker (i.e., C-reactive protein), physical and cognitive ability (i.e., gaits and episodic memory), and chronic conditions did not differ by subjective age. The findings indicate that reports of subjective age correlate with perceived rather than objective health status. Subjective age may therefore function as an indicator that health problems or life stressors are becoming internalized and overcoming an individual’s capacity to cope. Future research is needed to understand how subjective age can act as a bellweather of subjective and objective health decline.

